# Prostate Cancer Risks for Male *BRCA1* and *BRCA2* Mutation Carriers: A Prospective Cohort Study^☆^

**DOI:** 10.1016/j.eururo.2019.08.025

**Published:** 2020-01

**Authors:** Tommy Nyberg, Debra Frost, Daniel Barrowdale, D. Gareth Evans, Elizabeth Bancroft, Julian Adlard, Munaza Ahmed, Julian Barwell, Angela F. Brady, Carole Brewer, Jackie Cook, Rosemarie Davidson, Alan Donaldson, Jacqueline Eason, Helen Gregory, Alex Henderson, Louise Izatt, M. John Kennedy, Claire Miller, Patrick J. Morrison, Alex Murray, Kai-Ren Ong, Mary Porteous, Caroline Pottinger, Mark T. Rogers, Lucy Side, Katie Snape, Lisa Walker, Marc Tischkowitz, Rosalind Eeles, Douglas F. Easton, Antonis C. Antoniou

**Affiliations:** a Centre for Cancer Genetic Epidemiology, Department of Public Health and Primary Care, University of Cambridge, Cambridge, UK; b Manchester Regional Genetics Service, Central Manchester University Hospitals NHS Foundation Trust, Manchester, UK; c Oncogenetics Team, Division of Genetics and Epidemiology, The Institute of Cancer Research, London, UK; d Yorkshire Regional Genetics Service, Leeds Teaching Hospitals NHS Trust, Leeds, UK; e North East Thames Regional Genetics Service, Great Ormond Street Hospital for Children NHS Trust, London, UK; f Leicestershire Clinical Genetics Service, University Hospitals of Leicester NHS Trust, Leicester, UK; g North West Thames Regional Genetics Service, London North West University Healthcare NHS Trust, London, UK; h Peninsula Clinical Genetics Service, Royal Devon and Exeter NHS Foundation Trust, Exeter, UK; i North Trent Clinical Genetics Service, Sheffield Children’s NHS Foundation Trust, Sheffield, UK; j West of Scotland Regional Genetics Service, NHS Greater Glasgow and Clyde, Glasgow, UK; k South Western Regional Genetics Service, University Hospitals Bristol NHS Foundation Trust, Bristol, UK; l Nottingham Centre for Medical Genetics, Nottingham University Hospitals NHS Trust, Nottingham, UK; m North of Scotland Regional Genetics Service, NHS Grampian, Aberdeen, UK; n Northern Genetics Service, Newcastle upon Tyne Hospitals NHS Foundation Trust, Newcastle, UK; o South East Thames Regional Genetics Service, Guy’s and St Thomas’ NHS Foundation Trust, London, UK; p St. James’s Hospital, Dublin, Ireland; q National Centre for Medical Genetics, Dublin, Ireland; r Merseyside and Cheshire Clinical Genetics Service, Liverpool Women’s NHS Foundation Trust, Liverpool, UK; s Northern Ireland Regional Genetics Service, Belfast Health and Social Care Trust, Belfast, UK; t Medical Genetics Services for Wales, Abertawe Bro Morgannwg University Health Board, Swansea, UK; u West Midlands Regional Genetics Service, Birmingham Women’s and Children’s NHS Foundation Trust, Birmingham, UK; v South East of Scotland Regional Genetics Service, NHS Lothian, Edinburgh, UK; w Medical Genetics Services for Wales, Betsi Cadwaladr University Health Board, Bodelwyddan, UK; x All Wales Medical Genetics Service, NHS Wales, Cardiff, UK; y Wessex Clinical Genetics Service, University Hospital Southampton NHS Foundation Trust, Southampton, UK; z South West Thames Regional Genetics Service, St George’s University Hospitals NHS Foundation Trust, London, UK; A Oxford Regional Genetics Service, Oxford University Hospitals NHS Foundation Trust, Oxford, UK; B Department of Medical Genetics, University of Cambridge, Cambridge, UK; C East Anglian Regional Genetics Service, Cambridge University Hospitals NHS Trust, Cambridge, UK; D Cancer Genetics Unit, Royal Marsden NHS Foundation Trust, London, UK

**Keywords:** *BRCA1*, *BRCA2*, Genetic risk, Prospective cohort study, Prostate cancer

## Abstract

**Background:**

*BRCA1* and *BRCA2* mutations have been associated with prostate cancer (PCa) risk but a wide range of risk estimates have been reported that are based on retrospective studies.

**Objective:**

To estimate relative and absolute PCa risks associated with *BRCA1/2* mutations and to assess risk modification by age, family history, and mutation location.

**Design, setting, and participants:**

This was a prospective cohort study of male *BRCA1* (*n* = 376) and *BRCA2* carriers (*n* = 447) identified in clinical genetics centres in the UK and Ireland (median follow-up 5.9 and 5.3 yr, respectively).

**Outcome measurements and statistical analysis:**

Standardised incidence/mortality ratios (SIRs/SMRs) relative to population incidences or mortality rates, absolute risks, and hazard ratios (HRs) were estimated using cohort and survival analysis methods.

**Results and limitations:**

Sixteen *BRCA1* and 26 *BRCA2* carriers were diagnosed with PCa during follow-up. *BRCA2* carriers had an SIR of 4.45 (95% confidence interval [CI] 2.99–6.61) and absolute PCa risk of 27% (95% CI 17–41%) and 60% (95% CI 43–78%) by ages 75 and 85 yr, respectively. For *BRCA1* carriers, the overall SIR was 2.35 (95% CI 1.43–3.88); the corresponding SIR at age <65 yr was 3.57 (95% CI 1.68–7.58). However, the *BRCA1* SIR varied between 0.74 and 2.83 in sensitivity analyses to assess potential screening effects. PCa risk for *BRCA2* carriers increased with family history (HR per affected relative 1.68, 95% CI 0.99–2.85). *BRCA2* mutations in the region bounded by positions c.2831 and c.6401 were associated with an SIR of 2.46 (95% CI 1.07–5.64) compared to population incidences, corresponding to lower PCa risk (HR 0.37, 95% CI 0.14–0.96) than for mutations outside the region. *BRCA2* carriers had a stronger association with Gleason score ≥7 (SIR 5.07, 95% CI 3.20–8.02) than Gleason score ≤6 PCa (SIR 3.03, 95% CI 1.24–7.44), and a higher risk of death from PCa (SMR 3.85, 95% CI 1.44–10.3). Limitations include potential screening effects for these known mutation carriers; however, the *BRCA2* results were robust to multiple sensitivity analyses.

**Conclusions:**

The results substantiate PCa risk patterns indicated by retrospective analyses for *BRCA2* carriers, including further evidence of association with aggressive PCa, and give some support for a weaker association in *BRCA1* carriers.

**Patient summary:**

In this study we followed unaffected men known to carry mutations in the *BRCA1* and *BRCA2* genes to investigate whether they are at higher risk of developing prostate cancer compared to the general population. We found that carriers of *BRCA2* mutations have a high risk of developing prostate cancer, particularly more aggressive prostate cancer, and that this risk varies by family history of prostate cancer and the location of the mutation within the gene.

## Take Home Message

Male *BRCA2* mutation carriers have a high risk of developing prostate cancer, particularly high-grade disease. However, the risk is heterogeneous and varies with family history and the location of the mutation within the gene. *BRCA1* mutations may have a similar but weaker association.

## 1 Introduction

Deleterious mutations in the tumour suppressor genes *BRCA1* and *BRCA2* are associated with high risks of breast and ovarian cancer [1], [2], and have been implicated in the genetic susceptibility to prostate cancer (PCa). Retrospective studies have reported that *BRCA2* mutations are associated with relative risks (RRs) of PCa in the range 2–6 [3], [4], [5], [6], [7], [8], [9], [10], [11], [12], [13]. RR estimates reported were higher for younger ages, in the range 6–9 for those aged <65 yr [4], [6], [13], [14], [15], and *BRCA2* carriers present more often with aggressive PCa [8], [9]. The evidence of association between *BRCA1* mutations and PCa risk is inconsistent, with reported RRs in the range 0.3–4 [3], [5], [7], [8], [9], [10], [12], [13], [16], [17], [18], [19], [20]. A recent meta-analysis reported a moderate association between *BRCA1* mutations and PCa risk (pooled odds ratio 1.35, 95% CI 1.03–1.76) [3], but two studies have reported RRs of 2–4 for *BRCA1* carriers younger than 65 yr [17], [21]. Studies have also reported variation in PCa risk by mutation location and type [6], [8], [10], [13], [22], [23].

There are only a few estimates of absolute risks of PCa for *BRCA1/2* mutation carriers and those are based on retrospective studies [4], [6], [7], [13], [15], [17], [21], [22]. Given the rapidly rising incidence of PCa in the prostate-specific antigen (PSA) testing era, retrospective absolute risk estimates may not be representative of the risks for mutation carriers currently seen in genetics clinics. Only two small prospective cohort studies of male *BRCA1/2* carriers have been reported [12], [24], the largest of which followed 137 *BRCA1* and 71 *BRCA2* carriers for an average of 5.1 yr, and did not show an association with PCa [24].

In the present study, we report age-specific PCa risk estimates for a large prospective cohort of male *BRCA1* and *BRCA2* carriers. We present relative and absolute risks, investigate variability in these risks by family history and mutation location, and consider the risk of developing high-grade PCa.

## 2 Patients and methods

### 2.1 EMBRACE study participants

EMBRACE (http://ccge.medschl.cam.ac.uk/embrace/) is a cohort study of *BRCA1* and *BRCA2* mutation carriers initiated in 1998. Participants were recruited via clinical genetics centres across the UK and Ireland, and were counselled with regard to their mutation status. This analysis included all male participants without a PCa diagnosis at recruitment who carried mutations considered to be pathogenic on the basis of widely accepted criteria (ENIGMA consortium; https://enigmaconsortium.org/). All participants completed a baseline questionnaire that included information on known and suspected cancer risk factors, medical history, and personal and family cancer history. Follow-up data were collected through linkage with national registers covering England, Wales, and Scotland, and questionnaires that were collected at 2, 5, and 10 yr after baseline. For self-reported cancers, confirmation was sought from the participating clinics. For the present study, the end of follow-up was set as June 30, 2016 to ensure that cancer diagnoses were likely to have been reported at the time of the last record linkage (performed on October 4, 2016) or as the date of the last questionnaire returned if one was available after June 30, 2016.

All participants provided written informed consent. The study was approved by the Anglia and Oxford Medical Research and Ethics Committee.

### 2.2 Statistical analysis

We prospectively followed the participants from completion of their baseline questionnaire until their age at diagnosis of PCa, age of death, age at the end-of-follow-up, or age 85 yr, whichever occurred first. A diagnosis of another cancer or of prostatic intraepithelial neoplasia was not considered as a censoring event. Analogously, we followed the participants for deaths due to PCa.

We compared the observed PCa incidence and PCa mortality to those expected from population incidences and PCa-specific mortality rates (Office for National Statistics, https://www.ons.gov.uk/) using standardised incidence ratios (SIRs) or standardised mortality ratios (SMRs) computed via Poisson regression. We used the Kaplan-Meier estimator to estimate absolute risks, and Cox regression to test for differences in risk between subgroups.

We classified men who had at least one first- or second-degree relative diagnosed with PCa as having a positive PCa family history, and assessed trends in risks according to the number of affected relatives. We investigated differences in risk by mutation position using prespecified definitions of regions for which different associations with PCa risk have been identified in published studies [6], [10], [13], [22], [23]. To assess the association of *BRCA1/2* mutations with clinical PCa subtypes according to biopsy Gleason score (GS), we compared the observed number of PCa diagnoses by GS subtype to those expected given population GS-specific incidences. We used competing risk estimators to estimate the absolute risk for these clinical subtypes. Because data on GS were not available for all PCas, we used multiple imputation to avoid omission of PCa events.

For the main analysis, we included men with previous non-prostate cancers, did not censor for non-prostate cancers during follow-up, and considered follow-up up to the last questionnaire if available after the last record linkage. We assessed the impact of these assumptions in sensitivity analyses. We also repeated the analysis after omitting pathogenic missense mutations to assess the impact of such less clearly deleterious mutations.

Mutation carriers may be offered a different screening and diagnosis regimen than men in the general population [25]. We performed further analyses to assess the potential impact of such differential screening. First, we performed landmark analyses where follow-up was initiated at 6 or 12 mo after baseline. Second, on the basis of previous findings that observed PCa incidences are 1.4–1.9 times higher for men undergoing PSA screening at regular intervals in comparison to unscreened men [26], we estimated SIRs relative to population incidences multiplied by adjustment factors of 1.6 and 1.9. To obtain absolute risk estimates, we used weighted Kaplan-Meier estimators. Furthermore, in October 2005 the UK-based IMPACT screening trial started recruiting *BRCA1/2* carriers [27]. Although the exact overlap between the studies is unclear, to investigate the impact on risk estimates we assessed (1) PCa risks separately for participants from IMPACT-recruiting centres and their person-time from October 2005 and after; and (2) the person-time for participants from these centres before October 2005 in addition to the entire person-time for participants from non–IMPACT-recruiting centres.

Statistical analysis was performed using R (version 3.4.4; R Foundation for Statistical Computing, Vienna, Austria). Full details of all methods are given in the Supplementary material.

## 3 Results

### 3.1 Prostate cancer

In total, 16 of 376 *BRCA1* and 26 of 447 *BRCA2* mutation carriers were diagnosed with PCa during median follow-up of 5.9 and 5.3 yr, respectively (Table 1). All PCa diagnoses were confirmed via either registry linkage or the participating clinics.

**Table 1:** Participant characteristics.

Parameter	Result	
Initially recruited, *n*	998	
Excluded: mutation in both *BRCA1* and *BRCA2*	4	
	***BRCA1*****carriers**	***BRCA2*****carriers**
Initially recruited, *n*	451	543
Excluded: variant of unknown significance	3	3
Excluded: previous prostate cancer diagnosis	14	37
Excluded: age ≥85 yr at baseline	1	0
Excluded: no follow-up beyond baseline	57	56
**Men included,*****n***^a^	**376**	**447**
Year of study entry, *n* (%)		
1999–2004^b^	69 (18)	48 (11)
2005–2010	144 (38)	172 (38)
2011–2016	163 (43)	227 (51)
Median age at study entry, yr (interquartile range)	54.0 (43.2–64.1)	51.4 (41.5–63.6)
Age group at study entry, yr, *n* (%)		
19–44	103 (27)	155 (35)
45–54	97 (26)	105 (23)
55–64	96 (26)	102 (23)
65–74	65 (17)	66 (15)
75–83	15 (4.0)	19 (4.3)
Median follow-up, yr (interquartile range)^c^	5.9 (3.0–10.1)	5.3 (2.6–8.9)
Family history of prostate cancer, *n* (%)^d^		
No	297 (79)	328 (73)
Yes	48 (13)	87 (19)
Unknown (at least 1 male relative with unknown cancer site)	14 (3.7)	16 (3.6)
Missing data	17 (4.5)	16 (3.6)
Previous non–prostate cancer diagnosis, *n* (%)		
No	355 (94)	390 (87)
Yes^e^	21 (5.6)	57 (13)
Non–prostate cancer diagnosis during follow-up, *n* (%)		
No^f^	349 (93)	429 (96)
Yes^g^	27 (7.2)	18 (4.0)
**Prostate cancer diagnosis,*****n***	**16**	**26**
Median age at prostate cancer diagnosis, yr (interquartile range)	66.0 (61.9–71.7)	71.4 (62.8–77.5)
Diagnostic modality, *n* (%)		
Screening	11 (69)	14 (54)
Clinical symptoms	3 (19)	7 (27)
Missing data	2 (13)	5 (19)
Median PSA at diagnosis, ng/ml (interquartile range)	5.0 (3.6–5.9)	6.2 (4.3–21.6)
Clinical stage, *n* (%)		
T1	1 (6.3)	4 (15)
T2	7 (44)	12 (46)
T3	4 (25)	2 (7.7)
T4	0 (0)	1 (3.8)
TX	1 (6.3)	1 (3.8)
Missing data	3 (19)	6 (23)
Gleason score, *n* (%)		
≤6	7 (44)	4 (15)
3 + 4	4 (25)	7 (27)
4 + 3	0 (0)	3 (12)
≥8	2 (13)	5 (19)
Missing data	3 (19)	7 (27)

PSA = prostate-specific antigen.

a *BRCA1*: 309 singletons, 23 families with two relatives, four families with three relatives, one family with four relatives, and one family with five relatives. *BRCA2*: 353 singletons, 36 families with two relatives, six families with three relatives, and one family with four relatives.

b Study recruitment was initiated in August 1998 but the first male participant was recruited in February 1999.

c Calculated using the reverse Kaplan-Meier method.

d At least one first- or second-degree relative diagnosed with prostate cancer.

e Includes four *BRCA1* and 35 *BRCA2* carriers with male breast cancer.

f Includes three *BRCA2* carriers who were diagnosed with high-grade prostatic intraepithelial neoplasia and who did not develop any malignant tumours.

g Includes one *BRCA1* and two *BRCA2* carriers with male breast cancer, and two *BRCA1* and three *BRCA2* carriers with pancreatic cancer.

**Fig. 1:**
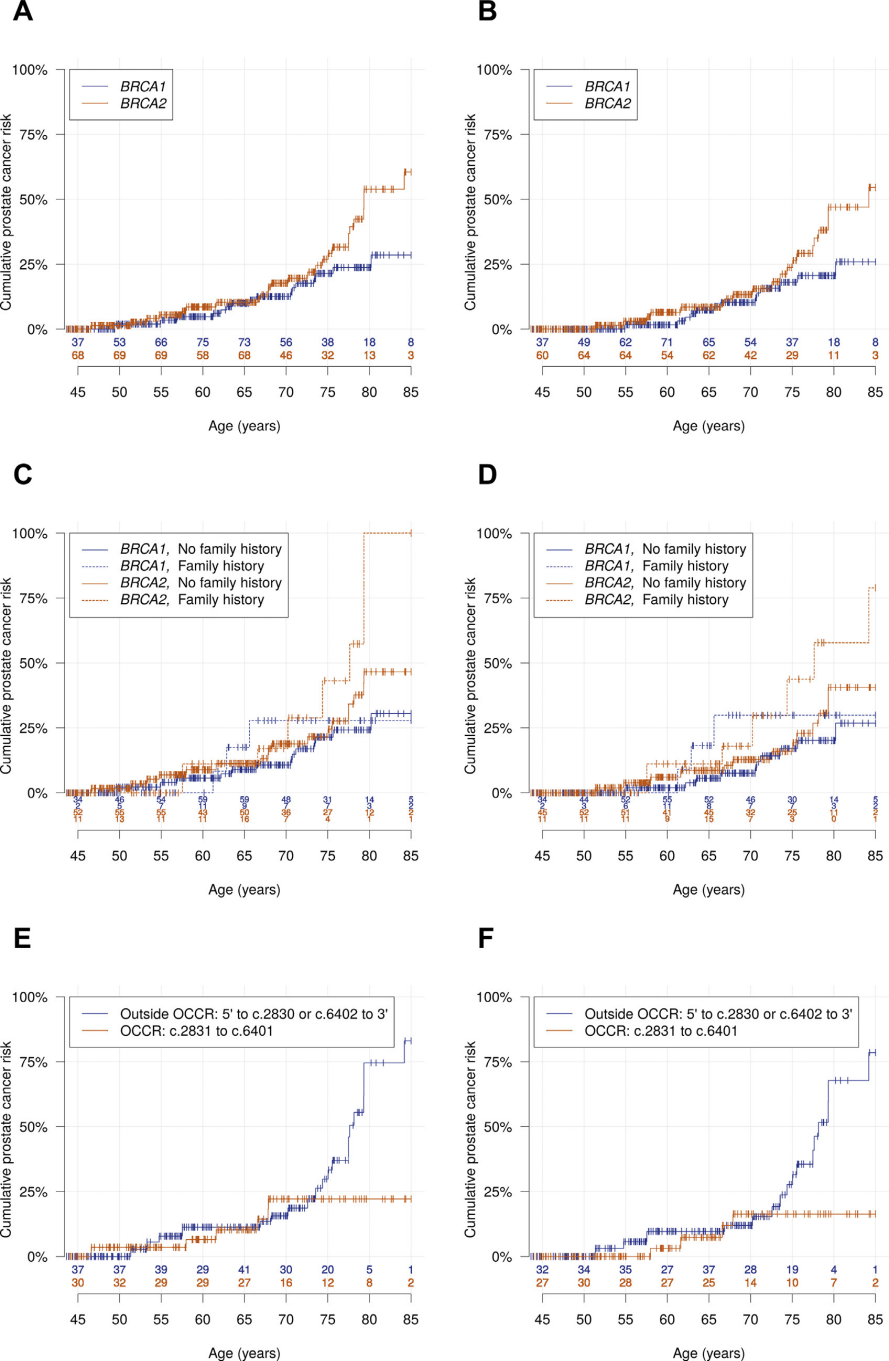
Absolute prostate cancer risk for *BRCA1* and *BRCA2* mutation carriers, with the number at risk at each age on the x-axis. (A) Overall; (B) overall, with follow-up initiated at 6 mo after study entry; (C) by family history; (D) by family history, with follow-up initiated at 6 mo after study entry; (E) by the *BRCA2* ovarian cancer cluster region (OCCR, wide definition) [2], [22]; and (F) by the *BRCA2* OCCR (wide definition) [2], [22] with follow-up initiated at 6 mo after study entry. Family history was defined as having at least one first- or second-degree relative with a prostate cancer diagnosis at the time of study entry.

Carrying a *BRCA1* mutation was associated with a PCa SIR of 2.35 (95% confidence interval [CI] 1.43–3.88) relative to the population incidence, whereas the SIR for *BRCA2* carriers was 4.45 (95% CI 2.99–6.61). For *BRCA1* carriers, the SIR was 3.57 (95% CI 1.68–7.58) for ages <65 yr and 1.86 (95% CI 0.96–3.59) for ages ≥65 yr. The SIR estimates by age were similar for *BRCA2* carriers (3.99, 95% CI 1.88–8.49 for <65 yr; 4.64, 95% CI 2.91–7.41 for ≥65 yr). The estimated absolute risk of PCa was 21% (95% CI 13–34%) by age 75 yr and 29% (95% CI 17–45%) by age 85 yr for *BRCA1* carriers. The corresponding PCa risks for *BRCA2* carriers were 27% (95% CI 17–41%) and 60% (95% CI 43–78%), respectively (Table 2; Fig. 1A and 1B).

**Table 2:** SIRs and absolute PCa risks for *BRCA1* and *BRCA2* mutation carriers overall and by age and PCa FH.

Gene	Group	*n*	PYs	OEs	IR per 1000 PY (95% CI)	EEs	SIR (95% CI)	Cumulative PCa risk, % (95% CI)^a^
**Overall**								
*BRCA1*	Age 19–44 yr	103	510.0	0	0.00	0.00	0.00	0
	Age 45–54 yr	134	556.0	2	3.60 (0.90–14.4)	0.21	9.56 (2.39–38.2)	3.5 (0.87–13)
	Age 55–64 yr	162	707.3	5	7.07 (2.92–17.1)	1.75	2.86 (1.18–6.94)	9.9 (4.8–20)
	Age 65–74 yr	138	539.1	7	13.0 (6.15–27.4)	3.32	2.11 (1.00–4.46)	21 (13–34)
	Age 75–84 yr	53	192.9	2	10.4 (2.57–41.9)	1.51	1.32 (0.33–5.33)	29 (17–45)
	Age 19–64 yr	296	1773.3	7	3.95 (1.88–8.31)	1.96	3.57 (1.68–7.58)	10 (4.8–20)
	Age 65–84 yr	153	731.9	9	12.3 (6.39–23.7)	4.84	1.86 (0.96–3.59)	29 (17–45)
	Overall	376	2505.3	16	6.39 (3.91–10.4)	6.80	2.35 (1.43–3.88)	29 (17–45)
*BRCA2*	Age 19–44 yr	155	622.9	0	0.00	0.01	0.00	0
	Age 45–54 yr	173	720.1	4	5.56 (2.05–15.0)	0.27	14.7 (5.43–39.8)	5.4 (2.1–14)
	Age 55–64 yr	171	593.2	3	5.06 (1.63–15.7)	1.47	2.04 (0.65–6.36)	10 (5.0–21)
	Age 65–74 yr	134	463.3	9	19.4 (9.93–38.0)	2.88	3.13 (1.60–6.12)	27 (17–41)
	Age 75–84 yr	51	155.0	10	64.5 (33.2–125.4)	1.21	8.25 (4.25–16.0)	60 (43–78)
	Age 19–64 yr	362	1936.2	7	3.62 (1.71–7.65)	1.75	3.99 (1.88–8.49)	10 (5.0–21)
	Age 65–84 yr	153	618.2	19	30.7 (19.3–49.0)	4.09	4.64 (2.91–7.41)	60 (43–78)
	Overall	447	2554.4	26	10.2 (6.92–15.0)	5.85	4.45 (2.99–6.61)	60 (43–78)
**By PCa FH**^b^								
*BRCA1*	No FH	311	2110.0	13	6.16 (3.58–10.6)	5.55	2.34 (1.35–4.07)	31 (17–50)
	FH	48	264.8	3	11.3 (3.54–36.3)	0.95	3.17 (0.97–10.4)^c^	28 (9.8–64)
*BRCA2*	No FH	344	1969.9	18	9.14 (5.75–14.5)	4.65	3.87 (2.40–6.23)	47 (31–65)
	FH	87	481.4	7	14.5 (6.78–31.2)	0.96	7.31 (3.40–15.7)^d^	–e

CI = confidence interval; EEs = expected events; FH = family history; HR = hazard ratio; IR = incidence rate; OEs = observed events; PCa = prostate cancer; PY = person-year; SIR = standardised incidence ratio.

a Kaplan-Meier estimated cumulative PCa risk by the end of each age interval or age 85 yr.

b At least one first- or second-degree relative diagnosed with PCa.

c *BRCA1* carriers: HR per affected first- or second-degree relative 1.33 (95% CI 0.42–4.20).

d *BRCA2* carriers: HR per affected first- or second-degree relative 1.68 (95% CI 0.99–2.85).

e PCa risk estimate for age 85 yr not available because of the low number of individuals left in follow-up. At age 75 yr, the cumulative PCa risk estimate was 43% (95% CI 18–80) for *BRCA2* carriers with a PCa FH and 22% (95% CI 12–36) for *BRCA2* carriers without a PCa FH.

For men with a positive family history, the SIR was 3.17 (95% CI 0.97–10.37) for *BRCA1* and 7.31 (95% CI 3.40–15.72) for *BRCA2* carriers. The corresponding SIRs for carriers without a family history were 2.34 (95% CI 1.35–4.07) and 3.87 (95% CI 2.40–6.23), respectively. For *BRCA2* carriers, the hazard ratio (HR) per affected relative was 1.68 (95% CI 0.99–2.85; Table 2; Fig. 1C and 1D).

Men with *BRCA2* mutations located in the central region of the gene (c.2831–c.6401; ovarian cancer cluster region [OCCR], wide definition [2], [22]; see Supplementary material online) had a significantly lower risk of PCa than men with mutations outside this region (HR 0.37, 95% CI 0.14–0.96). However, mutations both within (SIR 2.46, 95% CI 1.07–5.64) and outside (SIR 5.88, 95% CI 3.75–9.22) the OCCR were associated with higher than population PCa risk. When *BRCA2* mutations were grouped according to the narrow definition of the OCCR (c.3847–c.6275) [2], [22] the difference in PCa risk for mutations within and outside the OCCR was attenuated (HR 0.42, 95% CI 0.16–1.09; Table 3). The proportional hazards assumption was violated for this model (Schoenfeld residuals test, *p* =  0.005); the corresponding Kaplan-Meier curves revealed that the risks were similar between OCCR and non-OCCR mutation carriers at younger ages but deviated at older ages (Fig. 1E and 1F). The difference in risk between OCCR and non-OCCR mutation carriers (wide definition) was not statistically significant but was of similar magnitude after adjusting for family history (adjusted HR 0.40, 95% CI 0.15–1.07) and after omitting Ashkenazi mutation carriers (HR 0.43, 95% CI 0.15–1.24; Table 3).

**Table 3:** SIRs and absolute PCa risks for *BRCA2* mutation carriers by mutation location within the *BRCA2* gene.

Mutation location	*n*	PYs	OEs	IR per 1000 PY (95% CI)	EEs	SIR (95% CI)	Cumulative PCa risk, % (95% CI)^a^	Hazard ratio (95% confidence interval)		
								Unadjusted	Adjusted for FH	Excluding AFMCs^b^
***BRCA2* OCCR, wide definition**[2], [22]										
5′ to c.2830 or c.6402 to 3′ (non-OCCR)	267	1489.2	20	13.4 (8.64–20.9)	3.40	5.88 (3.75–9.22)	11 (4.3–28)	Reference	Reference	Reference
							30 (17–49)			
							83 (61–96)			
c.2831 to c.6401 (OCCR)	178	1054.4	6	5.69 (2.54–12.8)	2.44	2.46 (1.07–5.64)	10 (3.4–29)	0.37 (0.14-0.96)	0.40 (0.15-1.07)	0.43 (0.15-1.24)
							22 (11–43)			
							22 (11–43)			
Indeterminable	2									
***BRCA2* OCCR, narrow definition**[2], [22]										
5′ to c.3846 or c.6276 to 3′ (non-OCCR)	284	1581.8	20	12.6 (8.14–19.7)	3.56	5.62 (3.59–8.81)	10 (4.0–26)	Reference	Reference	Reference
							29 (16–48)			
							80 (59–94)			
c.3847 to c.6275 (OCCR)	161	961.8	6	6.24 (2.78–14.0)	2.28	2.63 (1.14–6.04)	11 (3.7–31)	0.42 (0.16-1.09)	0.46 (0.17-1.22)	0.50 (0.17-1.45)
							23 (11–45)			
							23 (11–45)			
Indeterminable	2									
***BRCA2* PCCR**[13]										
5′ to c.6372 or c.6493 to 3′ (non-PCCR)	444	2540.0	26	10.2 (6.95–15.1)	5.83	4.46 (3.00–6.64)	10 (5.0–21)	Reference		
							27 (17–41)			
							61 (43–79)			
c.6373 to c.6492 (PCCR)	3	14.4	0	0.00	0.02	0.00	0	Not done		
							0			
							0			

AFMCs = Ashkenazi founder mutation carriers; CI = confidence interval; EEs = expected events; FH = family history; HR = hazard ratio; IR = incidence rate; OCCR = ovarian cancer cluster region; OEs = observed events; PCa = prostate cancer; PCCR = PCa cluster region; PY = person-year; SIR = standardised incidence ratio.

a Kaplan-Meier estimated cumulative PCa risk by ages 65, 75, and 85 yr, respectively.

b Carriers of c.5946delT.

### 3.2 GS-specific PCa

For *BRCA1* carriers, the SIR was higher for GS ≤ 6 (SIR 3.50, 95% CI 1.67–7.35) than for GS ≥ 7 PCa (SIR 1.80, 95% CI 0.89–3.65). By contrast, for *BRCA2* carriers the SIR was higher for GS ≥ 7 (SIR 5.07, 95% CI 3.20–8.02) than for GS ≤ 6 PCa (SIR 3.03, 95% CI 1.24–7.44; Table 4). By age 85 yr, the absolute risk was 12% (95% CI 5.0–23%) for GS ≤ 6 and 16% (95% CI 6.4–30%) for GS ≥ 7 PCa among *BRCA1* carriers, and 9.3% (95% CI 2.9–20%) for GS ≤ 6 and 51% (95% CI 30%-69%) for GS ≥ 7 PCa among *BRCA2* carriers.

**Table 4:** GS-specific PCa SIRs for *BRCA1* and *BRCA2* mutation carriers.

Gene	*n*	PYs	Events with unknown GS	GS	OEs	EEs	SIR (95% confidence interval)		
							Without imputations	MIs^a^	MIs and 6-mo landmark
*BRCA1*	373	2488.9	3						
				≤6	7	2.19	3.25 (1.54–6.88)	3.50 (1.67–7.35)	2.26 (0.86–5.91)
				≥7	6	4.61	1.32 (0.59–2.98)	1.80 (0.89–3.65)	1.90 (0.93–3.85)
*BRCA2*	440	2537.4	7						
				≤6	4	1.83	2.23 (0.83–5.97)	3.03 (1.24–7.44)	2.01 (0.60–6.80)
				≥7	15	4.02	3.80 (2.27–6.38)	5.07 (3.20–8.02)	4.39 (2.63–7.31)

EEs = expected events; GS = Gleason score; MIs = multiple imputations; OEs = observed events; PCa = prostate cancer; PY = person-year; SIR = standardised incidence ratio.

a Pooled estimates from 100 imputations using multivariate imputation by chained equations. The following covariates were used for the imputation: PCa status, GS, prostate-specific antigen at diagnosis, clinical stage, diagnostic modality (screening/clinical), mutation gene (*BRCA1/2*), year of birth, age at study entry, age at follow-up, and family history (number of affected first- and second-degree relatives).

### 3.3 PCa mortality

Two *BRCA1* and four *BRCA2* carriers died from their incident PCa during follow-up. Compared to population PCa-specific mortality rates, the SMR was 1.75 (95% CI 0.44–6.90) for *BRCA1* and 3.85 (95% CI 1.44–10.3) for *BRCA2* carriers.

### 3.4 Sensitivity analyses

The estimated SIRs remained similar under alternative inclusion or censoring assumptions (Table 5). Of the 42 PCa diagnoses, nine occurred within the first 6 mo after study entry (Supplementary Table 1). In the landmark analyses, where follow-up was initiated at 6 or 12 mo after study entry, SIRs were lower for both *BRCA1* (6-mo landmark: SIR 2.02, 95% CI 1.17–3.50; 12-mo landmark: SIR 2.15, 95% CI 1.24–3.73) and *BRCA2* carriers (6-mo landmark: SIR 3.68, 95% CI 2.35–5.75; 12-mo landmark: SIR 3.37, 95% CI 2.08–5.47) but remained statistically significant. In the 6-mo landmark analysis, the estimated absolute PCa risk by age 85 yr was 26% (95% CI 15–43%) for *BRCA1* and 55% (95% CI 36–75%) for *BRCA2* carriers. When compared to a hypothetical population with higher PCa incidence, the association remained significant for *BRCA2* carriers (adjustment factor 1.9: SIR 2.34, 95% CI 1.57–3.48). The overall association was not significant for *BRCA1* carriers (adjustment factor 1.9: SIR 1.24, 95% CI 0.75–2.04), but the association for ages <65 yr remained significant with the lower adjustment factor of 1.6 (SIR 2.23, 95% CI 1.05–4.73). The corresponding absolute risk by age 85 yr when adjusted by a factor of 1.9 was 17% (95% CI 8–26%) for *BRCA1* and 41% (95% CI 22–59%) for *BRCA2* mutation carriers. When the landmark analysis was applied assuming higher population incidences, only the overall association between *BRCA2* mutations and PCa risk remained significant (SIR 2.30, 95% CI 1.47–3.60; Table 5).

**Table 5:** Sensitivity analyses.

Sensitivity analysis	Gene	Group	*n*	PYs	OEs	IR per 1000 PY (95% CI)	EEs	SIR (95% CI)	Cumulative PCa risk, % (95% CI)^a^
Excluding men with previous non-PCas	*BRCA1*	Age 19–64 yr	286	1724.5	7	4.06 (1.91–8.61)	1.90	3.68 (1.73–7.81)	10 (5.0–20)
		Age 65–84 yr	141	659.6	9	13.6 (7.09–26.2)	4.32	2.08 (1.08–4.01)	32 (19–50)
		Overall	355	2384.1	16	6.71 (4.09–11.0)	6.23	2.57 (1.56–4.23)	32 (19–50)
	*BRCA2*	Age 19–64 yr	342	1859.8	7	3.76 (1.78–7.96)	1.62	4.32 (2.03–9.21)	11 (5.3–22)
		Age 65–84 yr	110	454.0	11	24.2 (13.3–44.3)	2.95	3.72 (2.03–6.82)	60 (34–87)
		Overall	390	2313.8	18	7.78 (4.90–12.4)	4.57	3.94 (2.45–6.32)	60 (34–87)
Censoring for non-PCas in follow-up	*BRCA1*	Age 19–64 yr	296	1740.9	7	4.02 (1.91–8.46)	1.90	3.68 (1.74–7.81)	10 (5.0–20)
		Age 65–84 yr	151	684.5	9	13.1 (6.84–25.3)	4.51	2.00 (1.04–3.85)	30 (18–48)
		Overall	376	2425.4	16	6.60 (4.04–10.8)	6.41	2.50 (1.52–4.11)	30 (18–48)
	*BRCA2*	Age 19–64 yr	362	1919.4	7	3.65 (1.72–7.71)	1.73	4.06 (1.91–8.63)	10 (5.1–21)
		Age 65–84 yr	150	599.8	18	30.0 (18.6–48.5)	3.97	4.53 (2.80–7.32)	59 (42–78)
		Overall	447	2519.2	25	9.92 (6.69–14.7)	5.70	4.39 (2.93–6.57)	59 (42–78)
Censoring all on June 30, 2016	*BRCA1*	Age 19–64 yr	296	1751.7	7	4.00 (1.90–8.41)	1.92	3.64 (1.72–7.72)	10 (4.9–20)
		Age 65–84 yr	148	713.0	8	11.2 (5.62–22.4)	4.71	1.70 (0.85–3.40)	28 (17–44)
		Overall	376	2464.7	15	6.09 (3.67–10.1)	6.64	2.26 (1.35–3.78)	28 (17–44)
	*BRCA2*	Age 19–64 yr	362	1895.7	7	3.69 (1.75–7.81)	1.71	4.10 (1.93–8.74)	10 (5.1–21)
		Age 65–84 yr	153	599.7	19	31.7 (19.9–50.6)	3.97	4.79 (3.00–7.65)	61 (43–79)
		Overall	447	2495.4	26	10.4 (7.08–15.3)	5.67	4.58 (3.08–6.82)	61 (43–79)
Excluding missense mutation carriers	*BRCA1*	Age 19–64 yr	288	1741.0	7	4.02 (1.91–8.46)	1.94	3.61 (1.70–7.65)	10 (4.9–20)
		Age 65–84 yr	152	721.5	9	12.5 (6.48–24.0)	4.77	1.89 (0.98–3.64)	29 (18–45)
		Overall	368	2462.5	16	6.50 (3.97–10.6)	6.71	2.38 (1.45–3.93)	29 (18–45)
	*BRCA2*	Age 19–64 yr	358	1924.2	7	3.64 (1.72–7.69)	1.75	4.00 (1.88–8.50)	10 (5.0–21)
		Age 65–84 yr	148	593.8	18	30.3 (18.8–48.9)	3.91	4.60 (2.85–7.43)	61 (43–79)
		Overall	438	2517.9	25	9.93 (6.69–14.7)	5.67	4.41 (2.94–6.61)	61 (43–79)
Excluding Ashkenazi founder mutation carriers^b^	*BRCA1*	Age 19–64 yr	262	1535.8	5	3.26 (1.34–7.89)	1.64	3.05 (1.26–7.40)	8.2 (3.5–19)
		Age 65–84 yr	134	623.6	7	11.2 (5.34–23.6)	4.14	1.69 (0.80–3.56)	27 (15–47)
		Overall	332	2159.3	12	5.56 (3.15–9.81)	5.78	2.08 (1.17–3.68)	27 (15–47)
	*BRCA2*	Age 19–64 yr	330	1769.3	6	3.39 (1.51–7.62)	1.55	3.86 (1.71–8.72)	9.8 (4.5–21)
		Age 65–84 yr	136	533.0	19	35.6 (22.3–57.0)	3.53	5.38 (3.36–8.60)	65 (46–83)
		Overall	405	2302.4	25	10.9 (7.32–16.1)	5.09	4.91 (3.28–7.36)	65 (46–83)
Follow-up initiated 6 mo after baseline	*BRCA1*	Age 19–64 yr	268	1631.6	5	3.06 (1.27–7.42)	1.84	2.72 (1.12–6.58)	7.3 (3.1–17)
		Age 65–84 yr	149	691.7	8	11.6 (5.79–23.1)	4.59	1.74 (0.87–3.49)	26 (15–43)
		Overall	352	2323.3	13	5.60 (3.24–9.68)	6.43	2.02 (1.17–3.50)	26 (15–43)
	*BRCA2*	Age 19–64 yr	335	1761.7	5	2.84 (1.17–6.87)	1.61	3.10 (1.28–7.54)	8.5 (3.6–19)
		Age 65–84 yr	141	577.2	15	26.0 (15.5–43.7)	3.83	3.92 (2.33–6.60)	55 (36–75)
		Overall	414	2338.8	20	8.55 (5.51–13.3)	5.44	3.68 (2.35–5.75)	55 (36–75)
Follow-up initiated 12 mo after baseline	*BRCA1*	Age 19–64 yr	256	1500.4	5	3.33 (1.37–8.09)	1.73	2.89 (1.19–7.02)	7.8 (3.3–18)
		Age 65–84 yr	144	650.3	8	12.3 (6.14–24.6)	4.33	1.85 (0.92–3.71)	27 (15–45)
		Overall	341	2150.7	13	6.04 (3.49–10.5)	6.06	2.15 (1.24–3.73)	27 (15–45)
	*BRCA2*	Age 19–64 yr	313	1600.4	5	3.12 (1.29–7.57)	1.49	3.37 (1.38–8.21)	8.9 (3.8–20)
		Age 65–84 yr	136	535.7	12	22.4 (12.6–39.8)	3.56	3.37 (1.89–6.00)	51 (31–74)
		Overall	400	2136.1	17	7.96 (4.95–12.8)	5.05	3.37 (2.08–5.47)	51 (31–74)
Comparison to PI increased by a factor of 1.6 [26]]^c^	*BRCA1*	Age 19–64 yr	296	1773.3	7	3.95 (1.88–8.31)	3.14	2.23 (1.05–4.73)	6.3 (1.6–11)
		Age 65–84 yr	153	731.9	9	12.3 (6.39–23.7)	7.74	1.16 (0.60–2.24)	19 (8.8–30)
		Overall	376	2505.3	16	6.39 (3.91–10.4)	10.9	1.47 (0.89–2.42)	19 (8.8–30)
	*BRCA2*	Age 19–64 yr	362	1936.2	7	3.62 (1.71–7.65)	2.81	2.49 (1.17–5.31)	6.6 (1.7–11)
		Age 65–84 yr	153	618.2	19	30.7 (19.3–49.0)	6.55	2.90 (1.82–4.63)	46 (27–65)
		Overall	447	2554.4	26	10.2 (6.92–15.0)	9.35	2.78 (1.87–4.13)	46 (27–65)
Comparison to PI increased by a factor of 1.9 [26]^c^	*BRCA1*	Age 19–64 yr	296	1773.3	7	3.95 (1.88–8.31)	3.72	1.88 (0.89–3.99)	5.4 (1.6–9.3)
		Age 65–84 yr	153	731.9	9	12.3 (6.39–23.7)	9.19	0.98 (0.51–1.89)	17 (8.0–26)
		Overall	376	2505.3	16	6.39 (3.91–10.4)	12.9	1.24 (0.75–2.04)	17 (8.0–26)
	*BRCA2*	Age 19–64 yr	362	1936.2	7	3.62 (1.71–7.65)	3.33	2.10 (0.99–4.47)	5.6 (1.5–9.8)
		Age 65–84 yr	153	618.2	19	30.7 (19.3–49.0)	7.77	2.44 (1.53–3.90)	41 (22–59)
		Overall	447	2554.4	26	10.2 (6.92–15.0)	11.1	2.34 (1.57–3.48)	41 (22–59)
Follow-up initiated 6 mo after baseline, and comparison to PI increased by a factor of 1.6 [26]^c^	*BRCA1*	Age 19–64 yr	268	1631.6	5	3.06 (1.27–7.42)	2.94	1.70 (0.70–4.11)	4.8 (0.87–8.7)
		Age 65–84 yr	149	691.7	8	11.6 (5.79–23.1)	7.34	1.09 (0.54–2.18)	18 (7.1–28)
		Overall	352	2323.3	13	5.60 (3.24–9.68)	10.3	1.26 (0.73–2.19)	18 (7.1–28)
	*BRCA2*	Age 19–64 yr	335	1761.7	5	2.84 (1.17–6.87)	2.58	1.94 (0.80–4.72)	5.5 (0.67–10)
		Age 65–84 yr	141	577.2	15	26.0 (15.5–43.7)	6.12	2.45 (1.46–4.12)	40 (19–61)
		Overall	414	2338.8	20	8.55 (5.51–13.3)	8.70	2.30 (1.47–3.60)	40 (19–61)
All participants until October 1, 2005, and participants from centres not recruiting to IMPACT [27] after October 1, 2005	*BRCA1*	Age 19–64 yr	115	497.5	0	0.00	1.38	0.00	0
		Age 65–84 yr	54	208.3	2	9.60 (2.31–39.9)	3.07	1.05 (0.24–4.55)	11 (2.9–39)
		Overall	147	705.8	2	2.83 (0.69–11.6)	2.72	0.74 (0.18–3.04)	11 (2.9–39)
	*BRCA2*	Age 19–64 yr	113	439.7	3	6.82 (2.11–22.0)	0.67	6.75 (1.98–23.0)	20 (6.6–50)
		Age 65–84 yr	34	108.7	1	9.20 (1.27–66.7)	0.68	1.48 (0.20–10.7)	36 (13–75)
		Overall	134	548.4	4	7.29 (2.69–19.8)	1.12	3.57 (1.29–9.85)	36 (13–75)
Participants from centres recruiting to IMPACT [27] after October 1, 2005	*BRCA1*	Age 19–64 yr	241	1275.8	7	5.49 (2.61–11.5)	1.42	4.93 (2.33–10.4)	14 (6.7–26)
		Age 65–84 yr	120	523.7	7	13.4 (6.43–27.8)	3.52	1.99 (0.95–4.15)	34 (20–53)
		Overall	310	1799.5	14	7.78 (4.63–13.1)	4.94	2.83 (1.67–4.81)	34 (20–53)
	*BRCA2*	Age 19–64 yr	298	1496.4	4	2.67 (1.00–7.17)	1.42	2.81 (1.04–7.60)	7.7 (2.9–19)
		Age 65–84 yr	129	509.5	18	35.3 (21.8–57.2)	3.42	5.27 (3.25–8.54)	62 (44–80)
		Overall	372	2006.0	22	11.0 (7.16–16.8)	4.84	4.54 (2.96–6.99)	62 (44–80)

CI = confidence interval; EEs = expected events; IR = incidence rate; OEs = observed events; PCa = prostate cancer; PI = population incidence; PY = person-year; SIR = standardised incidence ratio.

a Kaplan-Meier estimated cumulative prostate cancer risk by the end of each age interval or age 85 yr.

b *BRCA1*: c.68_69delAG and c.5266dupC; *BRCA2*: c.5946delT.

c The absolute risks were estimated using a Kaplan-Meier estimator weighted by the inverse of the adjustment factor for men with events.

When follow-up was restricted to the period before initiation of the IMPACT screening trial [27], in addition to the entire follow-up for participants from non–IMPACT-recruiting centres, there was no association with PCa risk for *BRCA1* carriers (SIR 0.74, 95% CI 0.18–3.04). However, this was based on a small sample size and the 95% CI overlapped with that for the estimate for *BRCA1* carriers from IMPACT-recruiting centres with follow-up after October 2005 (SIR 2.83, 95% CI 1.67–4.81). The point estimates were similar for *BRCA2* carriers followed without potential overlap with the IMPACT trial period and recruiting centres (SIR 3.57, 95% CI 1.29–9.85) and those whose follow-up potentially overlapped with IMPACT (SIR 4.54, 95% CI 2.96–6.99). The SIR for ages <65 yr for *BRCA2* carriers with no potential overlap with IMPACT was 6.75 (95% CI 1.98–23.0; Table 5).

When follow-up was initiated 6 mo after baseline, the SIRs for *BRCA1* carriers were similar for GS ≤ 6 (SIR 2.26, 95% CI 0.86–5.91) and GS ≥ 7 PCa (SIR 1.90, 95% CI 0.93–3.85), in contrast to the main results. The difference in GS-specific SIR estimates remained for *BRCA2* carriers (GS ≤ 6: SIR 2.01, 95% CI 0.60–6.80; GS ≥ 7: SIR 4.39, 95% CI 2.63–7.31; Table 4). On the basis of this analysis, the absolute risk by age 85 yr for *BRCA1* carriers was 7.8% (95% CI 2.2–18%) for GS ≤ 6 and 18% (95% CI 7.1–33%) for GS ≥ 7 PCa. For *BRCA2* carriers the corresponding risk was 7.1% (95% CI 1.4–19%) and 47% (95% CI 25–66%), respectively.

## 4 Discussion

We estimated the risk of PCa for male *BRCA1* and *BRCA2* mutation carriers using data from a large prospective cohort. The results substantiate previous reports from retrospective studies of a strong association between *BRCA2* mutations and PCa risk, and give some support for a similar but weaker association for mutations in the *BRCA1* gene, particularly at younger ages.

Depending on the assumptions, we found that *BRCA2* carriers are at a two to five times higher risk of PCa compared to men in the general population, which is consistent with previous RR estimates in the range 2–6 [3], [4], [5], [6], [7], [8], [9], [10], [11], [12], [13]. Our *BRCA2* RR estimates did not vary substantially with age, in contrast to previous studies suggesting higher RRs at younger ages [4], [6], [13], [14], [15]. However, the higher RR estimate at ages <65 yr for the subset of *BRCA2* carriers with no potential overlap with the IMPACT screening trial suggests that the similarities in associations by age might be due to potential screening effects. However, owing to the small number of events at younger ages, the precision of the estimates was low. In line with previous studies [3], [5], [7], [8], [9], [10], [12], [13], [16], [17], [18], [19], [20], [21], our findings indicate that *BRCA1* mutations are at most associated with a moderate PCa risk at younger ages, with RR estimates in the range 2–4 for ages <65 yr. The evidence for an association is weak at older ages, with our RR estimates varying between 1 and 2. Much larger studies are required to clarify the association between *BRCA1* mutations and PCa risk.

The estimated cumulative risk of developing PCa by age 85 yr was 29% (95% CI 17–45%) for *BRCA1* and 60% (95% CI 43–78%) for *BRCA2* carriers. However, absolute PCa risks depend on the screening regimen used, and the PCa risks were lower in analyses that assessed the impact of potentially prevalent cancers and the excess PCa risk among PSA-screened individuals. Although our RR estimates are similar to previous estimates, the absolute risk estimates from the present study are higher than estimates from retrospective studies. Previous absolute PCa risk estimates by ages 65–80 yr range from 3% to 9% for *BRCA1* carriers [7], [17], [21] and from 15% to 34% for *BRCA2* carriers [4], [6], [7], [13], [15], [22] (Supplementary Table 2). It is plausible that absolute risk estimates based on historical data are not representative of the absolute PCa risks for *BRCA1/2* carriers in the PSA testing era. Prospective risk estimates may be more informative for counselling current mutation carriers. Only two previous prospective studies on PCa risk for male *BRCA1/2* carriers have been reported and were limited by small sample sizes and wide CIs for their RR estimates, and neither presented absolute risk estimates. In a prospective cohort of 62 carriers from the USA, *BRCA2* mutations were associated with higher PCa risk (SIR 4.89, 95% CI 1.96–10.08) but there was no significant association for *BRCA1* carriers (SIR 3.81, 95% CI 0.77–11.13) [12]. An Israeli study observed only three prospective PCas in 210 unaffected *BRCA1/2* carriers (median follow-up 5.1 yr) and chose not to report a prospective RR estimate [24].

The results indicate that PCa risks for mutation carriers increase with the number of affected relatives, consistent with findings in the general population [28]. This is also consistent with the hypothesis that other familial factors modify PCa risks for mutation carriers, and with recent observations that common PCa susceptibility genetic variants [29] modify PCa risk for *BRCA1/2* carriers [30]. This emphasises the importance of considering family history and other risk-modifying genetic factors when counselling male *BRCA1/2* carriers. However, it is possible that mutation carriers with a family history of PCa are more likely to be screened or biopsied than mutation carriers without a PCa family history; this may also partly explain the higher risk observed.

We found that *BRCA1* carriers were at higher risk of GS ≤ 6 disease, but after omitting diagnoses in the initial 6 mo after study recruitment, the associations with high- and low-grade disease were similar. *BRCA1* carriers were not at a significantly higher risk of PCa mortality, although the CI for the SMR estimate was wide. A lack of association between *BRCA1* mutations and PCa grade is in line with published data [8], [9], and the higher SIR for GS ≤ 6 disease might reflect a higher propensity for diagnosing indolent low-grade tumours that would not have been detected in the absence of the discovery of a deleterious mutation. Conversely, our results suggest that *BRCA2* mutations are associated with a more aggressive PCa phenotype; the association was stronger for GS ≥ 7 than for GS ≤ 6 tumours. Furthermore, we observed a significant association between *BRCA2* mutations and PCa mortality. Associations with high-grade disease and PCa mortality are consistent with previous reports for *BRCA2* carriers [8], [9], and suggest that the *BRCA2* findings are less affected by screening effects.

*BRCA2* mutations both within and outside the OCCR were associated with elevated PCa risk. However, our results suggest that carriers of mutations within the OCCR are at comparatively lower risk than carriers of mutations outside the OCCR, consistent with previous findings [6], [22]. They are also consistent with reports of lower PCa risk for carriers of the *BRCA2* c.5946delT Ashkenazi Jewish founder mutation, which is located in the OCCR [23]. Conversely, the results are in contrast to a UK study that reported a HR of 2.92 (95% CI 1.54–5.54) for OCCR compared to non-OCCR mutations [10]. However, this study was based on a retrospective cohort of *BRCA2* carriers and their relatives and the analyses were not adjusted for the ascertainment process.

Strengths of our study include the nationwide recruitment of mutation carriers, which supports the generalisability of our findings. Furthermore, this is the largest prospective cohort of men with deleterious *BRCA1/*2 mutations to date, and the prospective study design allows for direct estimation of both relative and absolute risks. We have provided risk estimates by family history and mutation location.

Although this is the largest prospective study reported to date, the precision of our estimates is still limited by a moderate sample size and the number of incident PCas and PCa deaths. The results by GS are limited by potential inaccuracies in tumour grading based on biopsies; however, since mutation carriers were recruited through a UK-wide study and SIRs were computed relative to national GS-specific incidences (which will have similar inaccuracies), variability in pathological grading is unlikely to have resulted in a systematic bias. Other limitations include possible oversampling of men with a family history of PCa as a result of the recruitment through clinical genetics centres. While this allowed us to obtain estimates applicable to mutation carriers both with and without a family history, the overall risk might be somewhat overestimated compared to average *BRCA1/2* carriers in the population. In addition, known mutation carriers who undergo genetic counselling may receive enhanced screening compared to men from the general population. More specifically, during the study period the IMPACT screening trial [27] also recruited male *BRCA1/2* carriers, and therefore some overlap between IMPACT and EMBRACE is likely. Given the background prevalence of indolent PCas that are undetectable in the absence of screening [31] and our observed clustering of PCa diagnoses shortly after study entry, it is plausible that some of these PCas would not have been discovered in the absence of diagnostic measures taken as a result of the discovery of a mutation. When we initiated follow-up at 6 or 12 mo after study entry, the estimated RRs were attenuated for both *BRCA1* and *BRCA2* carriers, but remained statistically significant. Furthermore, known mutation carriers may undergo a different screening regimen over an extended period of time in comparison to men in the general population [25]. To assess this we compared the PCa incidence observed to that expected from population incidences adjusted by screening effect sizes estimated in the ERSPC trial [26]. The SIRs for *BRCA2* carriers remained significant, but the excess risk for *BRCA1* carriers was not consistently significant, and was significant only for ages <65 yr. This adjustment is limited by the assumption of a constant average screening effect on the population PCa incidences based on published ERSPC estimates [26]. The ERSPC data also suggest that the effect of screening may be time-dependent, with a probable decrease in screening effect sizes with time since initiation of screening [26]. This time dependence was not considered in our analysis and can result in potential overestimation of SIRs if the true effect of screening on population incidences is higher than the assumed average during the follow-up period. However, our adjustment used the highest published average PSA screening effect size from ERSPC, and assumes that no screening occurs in the general population, which is unlikely given the rates of opportunistic screening [32] and may result in an attenuation of the SIR estimates. After using both a 6-month landmark to control for the detection of prevalent PCas, and higher population incidences, the SIRs remained significant only for *BRCA2* carriers. However, these may represent extreme overadjustments. Finally, when we restricted the follow-up to centres and/or time periods not overlapping with the IMPACT recruitment, we found no association between *BRCA1* mutations and PCa risk. This might suggest that the association observed for *BRCA1* carriers is driven by screening-induced diagnoses of indolent tumours, but caution is needed in the interpretation as the sample size used for this subgroup analysis was small. By contrast, the strength of the association was similar for *BRCA2* carriers regardless of potential overlap with IMPACT. Assuming that clinically significant tumours are likely to be diagnosed regardless of screening regimen, this observation is consistent with the hypothesis that *BRCA2* mutations are associated with a risk of more aggressive disease. It provides further evidence that the association between *BRCA2* mutations and PCa risk is unlikely to be explained by screening effects.

## 5 Conclusions

This prospective analysis substantiates previous reports on the RR of PCa for *BRCA1* and *BRCA2* mutation carriers from retrospective studies, and provides direct estimates of absolute PCa risk by family history and mutation characteristics. The results will be informative in the counselling of men who carry *BRCA1* or *BRCA2* mutations.

  ***Author contributions***: Tommy Nyberg had full access to all the data in the study and takes responsibility for the integrity of the data and the accuracy of the data analysis.

*Study concept and design*: Antoniou, Nyberg, Easton.

*Acquisition of data*: Frost, Barrowdale, Bancroft, Easton, Eeles, Evans, Tischkowitz, Adlard, Ahmed, Barwell, Brady, Brewer, Cook, Davidson, Donaldson, Eason, Gregory, Henderson, Izatt, Kennedy, Miller, Morrison, Murray, Ong, Porteous, Pottinger, Rogers, Side, Snape, Walker.

Analysis and interpretation of data: Nyberg, Antoniou, Easton.

*Drafting of the manuscript*: Nyberg, Antoniou, Easton.

*Critical revision of the manuscript for important intellectual content*: Evans, Frost, Barrowdale, Bancroft, Eeles, Tischkowitz, Adlard, Ahmed, Barwell, Brady, Brewer, Cook, Davidson, Donaldson, Eason, Gregory, Henderson, Izatt, Kennedy, Miller, Morrison, Murray, Ong, Porteous, Pottinger, Rogers, Side, Snape, Walker.

*Statistical analysis*: Nyberg, Antoniou, Easton.

*Obtaining funding*: Easton, Antoniou, Eeles, Evans.

Administrative, technical, or material support: Frost, Barrowdale, Bancroft.

*Supervision*: Antoniou, Easton.

*Other*: None.

  ***Financial disclosures:*** Tommy Nyberg certifies that all conflicts of interest, including specific financial interests and relationships and affiliations relevant to the subject matter or materials discussed in the manuscript (eg, employment/affiliation, grants or funding, consultancies, honoraria, stock ownership or options, expert testimony, royalties, or patents filed, received, or pending), are the following: None.

  ***Funding/Support and role of the sponsor*:** This work was supported by Cancer Research UK grants C12292/A20861 and C12292/A22820. EMBRACE was supported by Cancer Research UK grants C1287/A23382 and C1287/A26886. D. Gareth Evans is supported by a National Institute for Health Research grant to the Biomedical Research Centre, Manchester (IS-BRC-1215-20007). Rosalind Eeles is supported by Cancer Research UK grant C5047/A8385 and National Institute for Health Research support to the Biomedical Research Centre at The Institute of Cancer Research and The Royal Marsden NHS Foundation Trust. The sponsors played no direct role in the study.

  Acknowledgments

We thank all the participants in the EMBRACE study.
