# Supplementary material for: Prostate Cancer Risks for Male BRCA1[image] and BRCA2 Mutation Carriers: A Prospective Cohort Study
Source: Eur Urol. 2020 Jan;77(1):24–35. doi: 10.1016/j.eururo.2019.08.025 (PMC6926480; doi:10.1016/j.eururo.2019.08.025)
Supplement: Supplementary file 1 [file mmc1.docx]

**Supplementary material**

**Full details on the statistical analysis**

We prospectively followed the participants from the completion of their baseline questionnaire until their age at diagnosis of prostate cancer, age of death, age at the end of follow-up, or age 85, whichever occurred first. A diagnosis of another cancer or of prostatic intraepithelial neoplasia were not considered as censoring events. In all analyses the event of interest was a prostate cancer diagnosis. We calculated the total follow-up time using the reverse Kaplan—Meier method [1].

We compared the observed prostate cancer incidences in study participants to those expected from population incidences (Cancer registration statistics, England, Office for National Statistics, https://www.ons.gov.uk/), using standardised incidence ratios (SIR) computed with Poisson regression. For this purpose we used age-and-calendar-period specific incidences (available in five-year age bands for individual years 1998 to 2016). Analyses were carried out separately for *BRCA1* and *BRCA2* mutation carriers. To estimate the absolute risks for mutation carriers, we used the counting process formulation of the Kaplan—Meier estimator to account for varying ages at study entry. Cox regression was used to test for differences in risk between subgroups of mutation carriers (e.g. by family history or mutation characteristics). We classified men who had at least one first- or second-degree family member diagnosed with PCa as having positive PCa family history, and assessed trends in risks with the number of affected first- and second-degree relatives.

Analogously, we followed the participants for prostate-cancer-specific mortality from the completion of their baseline questionnaire until their age at death due to prostate cancer, or age at death due to other causes, age at the end of follow-up, or age 85. We computed prostate-cancer-specific standardised mortality ratios (SMR) compared to age-and-calendar-period-specific population prostate cancer mortality rates (available in five-year age bands for individual years 1998 to 2016; Deaths registered in England and Wales, England and Wales, Office for National Statistics, https://www.ons.gov.uk/).

To investigate differences in risk by mutation characteristics, we grouped mutations on the basis of mutation position within the genes. The grouping was pre-specified, using previously published definitions of regions that have demonstrated different associations with PCa risk [2–7]. The mutation locations were specified using HGVS nomenclature (http://varnomen.hgvs.org/), using cDNA reference sequences NM_007294.3 (*BRCA1*) and NM_000059.3 (*BRCA2*) and reference genome hg18. In HGVS nomenclature, the nucleotide numbering is from the A of the ATG translation initiator codon. For deletions or insertions where the position was uncertain the change was assumed to have occurred at the most 3′ position. Specifically for *BRCA2*, we assessed differences between mutations in the central region of the gene, known as the ovarian cancer cluster region (OCCR) [2], and mutations outside this region. For this purpose we used both the wide definition (c.2831 to c.6401) [2,3,8] and the narrow definition (c.3847 to c.6275) of the OCCR [2,3,8]. Similarly, we compared risks for mutations within a recently proposed prostate cancer cluster region (PCCR): c.6373 to c.6492 [7], to mutations located outside this region. However, only three men had a mutation in this PCCR, and hence we could not assess the differences in risk on the basis of this definition. The analyses by mutation position were adjusted for family history of prostate cancer, and we assessed the impact of carriers of Ashkenazi founder mutations on the mutation location results by excluding this subgroup.

To account for the correlation between male relatives we used sandwich estimators based on family-specific clusters for the standard errors in all Poisson and Cox regression models [9]. We used the Schoenfeld residuals test to assess the Cox regression proportional hazards assumption.

We assessed the association of *BRCA1/2* mutations with clinical subtypes of PCa based on biopsy Gleason score (GS), by comparing the observed number of PCa diagnoses by GS subtypes to those expected given population GS-specific incidences. The GS-specific incidences were calculated using the age-and-calendar-period-specific population distribution of GSs (GS≤6 or ≥7; England, Public Health England, available in five-year age bands in three-calendar-year bands for 1995-2016). For the SIR calculations, diagnosis of a competing PCa subtype ended the follow-up without an event. To estimate absolute risks of these clinical subtypes, we used competing risk estimators [10]. Because data on GSs were not available in the EMBRACE study for all PCas, we used multiple imputation to avoid omission of PCa events.

Missing values were imputed using Multivariate Imputation by Chained Equations [11], based on the following variables: Prostate cancer status, Gleason score, PSA at diagnosis, Clinical stage, Diagnostic modality (screening/clinical), Mutation gene (*BRCA1/2*), Year of birth, Age at study entry, Age at follow-up, and Family history (number of affected first- and second-degree relatives). All variables were complete for all participants except for Family history, and tumour characteristics for 10 of the men with a known PCa. We used polytomous logistic regression to impute categorical variables and predictive mean matching for continuous variables. The imputation was repeated 100 times, and for the results by GS we present the resulting estimates after pooling the separate estimates calculated from each of the 100 repetitions.

For the main analysis, we allowed men with previous non-prostate cancers to be included, did not censor for non-prostate cancers during follow-up, and considered follow-up up to the last questionnaire if available after the last record linkage. We assessed the impact of these inclusion and censoring criteria by excluding men with previous non-prostate cancers, and in separate analyses by censoring men at the age of any non-prostate cancers (excluding non-melanoma skin cancers), or on 30th June 2016.

The analysis included carriers of missense mutations that have been classified as pathogenic based on the ENIGMA criteria (https://enigmaconsortium.org/); since such mutations may be associated with different risks than protein truncating mutations, we carried out a sensitivity analysis in which we omitted these missense mutations (eight *BRCA1* and nine *BRCA2* carriers).

Mutation carriers may be offered a different screening and diagnosis regimen than men in the general population [12]. Hypothetically, this might manifest in two ways: (1) early screening and detection of indolent PCas shortly after the detection of a mutation, and (2) enhanced screening for PCa over an extended period of time. To address this we performed a number of sensitivity analyses to assess the potential impact of such differential screening. Firstly, we performed landmark analyses where follow-up was initiated six or twelve months after baseline. Second, based on previous findings that observed PCa incidences are 1.4-1.9 times higher for men undergoing PSA screening at regular intervals as compared to unscreened men [13], we compared the observed PCa rates in our sample to population incidences multiplied by adjustment factors of 1.6 and 1.9. To obtain corresponding absolute risk estimates, we used weighted Kaplan—Meier estimators with bootstrap estimates for the standard errors (1000 repetitions). In this analysis, participants with incident PCa where assigned weights proportional to the inverse of the screening adjustment factor. Furthermore, in October 2005 the UK-based IMPACT screening trial started recruiting *BRCA1/2* participants [14]. Although the exact overlap between IMPACT and EMBRACE is unclear, we assessed PCa risks separately for the follow-up that was potentially overlapping with the IMPACT trial and the non-overlapping follow-up. For this, we separately considered the person-time from October 2005 of participants from centres that recruited to IMPACT, and the person-time of participants from IMPACT-recruiting centres before the initiation of IMPACT in addition to the entire person-time of participants from non-IMPACT-recruiting centres.

We used R software (version 3.4.4) [15] for the statistical analysis.

**References**

[1] Schemper M, Smith TL. A note on quantifying follow-up in studies of failure time. Contemp Clin Trials 1996;17:343–6.

[2] Gayther SA, Mangion J, Russell P, Seal S, Barfoot R, Ponder BA, et al. Variation of risks of breast and ovarian cancer associated with different germline mutations of the BRCA2 gene. Nat Genet 1997;15:103–5. doi:10.1038/ng0197-103.

[3] Thompson D, Easton D. Variation in cancer risks, by mutation position, in BRCA2 mutation carriers. Am J Hum Genet 2001;68:410–9. doi:10.1086/318181.

[4] Lubinski J, Phelan CM, Ghadirian P, Lynch HT, Garber J, Weber B, et al. Cancer variation associated with the position of the mutation in the BRCA2 gene. Fam Cancer 2004;3:1–10. doi:10.1023/B:FAME.0000026816.32400.45.

[5] van Asperen CJ, Brohet RM, Meijers-Heijboer EJ, Hoogerbrugge N, Verhoef S, Vasen HFA, et al. Cancer risks in BRCA2 families: Estimates for sites other than breast and ovary. J Med Genet 2005;42:711–9. doi:10.1136/jmg.2004.028829.

[6] Moran A, O’Hara C, Khan S, Shack L, Woodward E, Maher ER, et al. Risk of cancer other than breast or ovarian in individuals with BRCA1 and BRCA2 mutations. Fam Cancer 2012;11:235–42. doi:10.1007/s10689-011-9506-2.

[7] Roed Nielsen H, Petersen J, Therkildsen C, Skytte A-B, Nilbert M. Increased risk of male cancer and identification of a potential prostate cancer cluster region in BRCA2. Acta Oncol (Madr) 2016;55:38–44. doi:10.3109/0284186X.2015.1067714.

[8] Kuchenbaecker KB, Hopper JL, Barnes DR, Phillips K-A, Mooij TM, Roos-Blom M-J, et al. Risks of Breast, Ovarian, and Contralateral Breast Cancer for BRCA1 and BRCA2 Mutation Carriers. JAMA 2017;317:2402–16. doi:10.1001/jama.2017.7112.

[9] Zeileis A. Object-Oriented Computation of Sandwich Estimators. J Stat Softw 2006;16. doi:10.18637/jss.v016.i09.

[10] Aalen OO, Johansen S. An empirical transition matrix for non-homogeneous Markov chains based on censored observations. Scand J Stat 1978;5:141–50.

[11] van Buuren S, Groothuis-Oudshoorn K. mice : Multivariate Imputation by Chained Equations in R. J Stat Softw 2011;45. doi:10.18637/jss.v045.i03.

[12] National Comprehensive Cancer Network. NCCN Clinical Practice Guidelines in Oncology: Prostate Cancer Early Detection. Version 2.2018. 2018.

[13] Hugosson J, Roobol MJ, Månsson M, Tammela TLJ, Zappa M, Nelen V, et al. A 16-yr Follow-up of the European Randomized study of Screening for Prostate Cancer. Eur Urol 2019;76:43–51. doi:10.1016/j.eururo.2019.02.009.

[14] Bancroft EK, Page EC, Castro E, Lilja H, Vickers A, Sjoberg D, et al. Targeted prostate cancer screening in BRCA1 and BRCA2 mutation carriers: results from the initial screening round of the IMPACT study. Eur Urol 2014;66:489–99. doi:10.1016/j.eururo.2014.01.003.

[15] R Core Team. R: a language and environment for statistical computing 2018.
