# Supplementary material for: Prostate Cancer Risks for Male BRCA1[image] and BRCA2 Mutation Carriers: A Prospective Cohort Study
Source: Eur Urol. 2020 Jan;77(1):24–35. doi: 10.1016/j.eururo.2019.08.025 (PMC6926480; doi:10.1016/j.eururo.2019.08.025)
Supplement: Supplementary file 2 [file mmc2.pdf]

Supplementary table 1: Incidence rate by time since baseline.

Gene	Time since baseline	n	Person-years	Observed events	Incidence rate per 1000 person-years
BRCA1					
	0–6 mo	376	181.97	3	16.49
	6 mo–1 yr	352	172.61	0	0.00
	1–2 yr	341	337.00	2	5.93
	2–3 yr	334	302.46	1	3.31
	3–4 yr	279	262.75	5	19.03
	4–5 yr	251	240.08	1	4.17
	5–10 yr	231	725.81	4	5.51
	10–15 yr	94	247.95	0	0.00
BRCA2					
	0–6 mo	447	215.59	6	27.83
	6 mo–1 yr	414	202.69	3	14.80
	1–2 yr	400	389.08	4	10.28
	2–3 yr	381	332.76	4	12.02
	3–4 yr	303	278.21	5	17.97
	4–5 yr	257	249.17	0	0.00
	5–10 yr	241	710.77	3	4.22
	10–15 yr	87	163.59	1	6.11
