# Supplementary material for: Prostate Cancer Risks for Male BRCA1[image] and BRCA2 Mutation Carriers: A Prospective Cohort Study
Source: Eur Urol. 2020 Jan;77(1):24–35. doi: 10.1016/j.eururo.2019.08.025 (PMC6926480; doi:10.1016/j.eururo.2019.08.025)
Supplement: Supplementary file 3 [file mmc3.pdf]

Supplementary table 2: Published age-specific absolute prostate cancer risk estimates and 95% confidence intervals.

Gene	Publication	Study design	Setting	Age								
				45	50	55	60	65	70	75	80	85
BRCA1												
	Thompson, Easton and Breast Cancer Linkage Consortium, 2002 [1]	Retrospective, Case-family cohort	Europe		0.04% (0.03-0.06%)				2.64% (1.95-3.57%)			
			North America		0.12% (0.07-0.21%)				7.67% (4.77-12.20%)			
	Risch et al, 2006 [2]	Retrospective, Case-family cohort	Canada								7.4% (0.59-63%)	
	Leongamorn-lert et al, 2012 [3]	Retrospective, Descriptive case series compared to historical population estimates	UK					8.60%				
	Present analysis	Prospective, Cohort	UK and Republic of Ireland	0%	2.0% (0.28-13%)	3.5% (0.87-13%)	4.8% (1.6-14%)	9.9% (4.8-20%)	13% (6.7-23%)	21% (13-34%)	24% (15-37%)	29% (17-45%)
BRCA2												
	The Breast Cancer Linkage Consortium, 1999 [4]	Retrospective, Case-family cohort	Europe and North America		0.1% (0.1-0.2%)		1.6% (0.9-2.3%)		7.5% (5.7-9.3%)		19.8% (15.2-24.2%)	
	Thompson and Easton, 2001 [5]	Retrospective, Case-family cohort	Europe and North America								Non-OCCR mutations: 33.6% (25.1-44.1%) OCCR mutations: 19.2% (10.7-33.1%)	
	van Asperen et al, 2005 [6]	Retrospective, Cohort	The Netherlands		0.1% (0.0-0.5%)		0.8% (0.0-2.3%)		5.2% (1.7-8.7%)		17.3% (12.5-22.0%)	

Risch et al, 2006 [2]	Retrospective, Case-family cohort	Canada								31% (13-62%)	
Kote-Jarai et al, 2011 [7]	Retrospective, Descriptive case series compared to historical population estimates	UK					15%				
Roed Nielsen et al, 2016 [8]	Retrospective, Cohort	Denmark								18.8% (16.6-21.9%)	
Present analysis	Prospective, Cohort	UK and Republic of Ireland	0%	1.4% (0.19-9.2%)	5.4% (2.1-14%)	8.5% (3.9-18%)	10% (5.0-21%)	18% (10-29%)	27% (17-41%)	54% (39-71%)	60% (43-78%)

References, Supplementary table 2

[1] Thompson D, Easton DF, Breast Cancer Linkage Consortium. Cancer incidence in BRCA1 mutation carriers. J Natl Cancer Inst 2002;94:1358–65. doi:10.1093/jnci/94.18.1358.

[2] Risch HA, McLaughlin JR, Cole DEC, Rosen B, Bradley L, Fan I, et al. Population BRCA1 and BRCA2 Mutation Frequencies and Cancer Penetrances: A Kin–Cohort Study in Ontario, Canada. Cancer 2006;98:1694–706. doi:10.1093/jnci/djj465.

[3] Leongamornlert D, Mahmud N, Tymrakiewicz M, Saunders E, Dadaev T, Castro E, et al. Germline BRCA1 mutations increase prostate cancer risk. Br J Cancer 2012;106:1697–701. doi:10.1038/bjc.2012.146.

[4] Breast Cancer Linkage Consortium. Cancer risks in BRCA2 mutation carriers. J Natl Cancer Inst 1999;91:1310–6. doi:10.1093/jnci/91.15.1310.

[5] Thompson D, Easton D. Variation in cancer risks, by mutation position, in BRCA2 mutation carriers. Am J Hum Genet 2001;68:410–9. doi:10.1086/318181.

[6] van Asperen CJ, Brohet RM, Meijers-Heijboer EJ, Hoogerbrugge N, Verhoef S, Vasen HFA, et al. Cancer risks in BRCA2 families: Estimates for sites other than breast and ovary. J Med Genet 2005;42:711–9. doi:10.1136/jmg.2004.028829.

[7] Kote-Jarai Z, Leongamornlert D, Saunders E, Tymrakiewicz M, Castro E, Mahmud N, et al. BRCA2 is a moderate penetrance gene contributing to young-onset prostate cancer: Implications for genetic testing in prostate cancer patients. Br J Cancer 2011;105:1230–4. doi:10.1038/bjc.2011.383.

[8] Roed Nielsen H, Petersen J, Therkildsen C, Skytte A-B, Nilbert M. Increased risk of male cancer and identification of a potential prostate cancer cluster region in BRCA2. Acta Oncol (Madr) 2016;55:38–44. doi:10.3109/0284186X.2015.1067714.
